# Robotic total hip arthroplasty for fused hips in ankylosing spondylitis patients: Our experience with robotic arm technology

**DOI:** 10.1051/sicotj/2022024

**Published:** 2022-06-29

**Authors:** Ashish Singh, Kartheek Telagareddy, Purushotam Kumar, Sushil Singh

**Affiliations:** 1 Anup Institute of Orthopaedics and Rehabilitation G-75-77 PC Colony Kankarbagh Patna 800020 India

**Keywords:** Fused hips, Ankylosing spondylitis, Robotic total hip arthroplasty, Bony fusion

## Abstract

The conversion from a fused hip to conventional total hip arthroplasty (THA) in patients with ankylosing spondylitis can be challenging. The problems are related to patient positioning, surgical exposure, femoral neck osteotomy, identifying the true acetabulum, and proper implant positioning. This case series describes our experience using the Mako Robotic-Arm in four bilateral THA procedures (each conducted in a single session) and one unilateral procedure in a fifth patient. Robotic total hip arthroplasty (RTHA) simplified THA by providing real-time information on the relative positions of the femur, pelvis, instruments, and implants to guide the surgery and implant placement.

## Introduction

Total hip arthroplasty (THA) in ankylosed hips of patients with ankylosing spondylitis (AS) is a technically challenging procedure. The clinical outcomes are generally less satisfactory than those for routine THA performed for osteoarthritis and other conditions [1]. Most surgeons have limited clinical experience with these complex surgeries. Fusion breakdown is a considerable challenge, especially in cases of bilateral hip involvement. Ankylosis of the hip dramatically affects the gait, as well as the biomechanics and kinematics of the spine and adjacent joints. The most frequent indication of THA is the need for pain relief and functional improvement in relatively young individuals.

Researchers have implicated abnormal spino-pelvic biomechanics in the challenges regarding THA in AS patients. Difficulties include inpatient positioning, surgical exposure, femoral neck osteotomy, identifying the true acetabulum, and proper cup positioning [1]. In this case series, we present nine robotic total hip arthroplasty (RTHA) procedures in five patients: four bilateral procedures (each in a single session) and one unilateral procedure in the fifth patient. The Mako Robotic-Arm System (Mako Surgical Corp., Stryker, Fort Lauderdale, FL, USA), using Mako Express Workflow Version 3.0, was used to perform all RTHA procedures.

In conventional THA, preoperative planning regarding acetabular cup anteversion and abduction (based on the spino-pelvic relationship) to prevent impingement or dislocation is easy; however, execution is difficult. This study aimed to elucidate how a robotic arm can help the surgeon by providing real-time information on the relative position of the femur, pelvis, instruments, and implants in order to guide the surgery and implant placement.

## Case descriptions

Our Institutional Review Board approved this case series, and patients provided written informed consent for their information and images to be published. The five patients (age range: 17–42 years) who underwent RTHA were human leukocyte antigen (HLA)-B27 positive and had ankylosed hips. Nine RTHA procedures were conducted on these patients between August 2020 and May 2021. There were four bilateral cases (each in a single session) and one unilateral case.

A clinical examination was performed followed by routine radiographs and computed tomography (CT) scans of the pelvis, proximal femur, and knees. After CT scan segmentation, which led to the creation of a patient-specific virtual three-dimensional model of the native hip anatomy, CT-based planning was conducted. There was a significant deviation from the standard protocol (requiring authorization from the surgeon) as fused hips were being treated. Proximal femur and pelvis segmentation was conducted en bloc instead of separately.

All surgeries occurred under spinal anesthesia using the standard posterolateral approach. The mean operation time, mean registration time (from pelvic array placement to complete registration), as well as abduction and anteversion angles were assessed. Total blood loss was calculated using the equation reported by Good et al. [2]. Oral indomethacin (25 mg, 3 times daily) was prescribed for all patients for 6 weeks as heterotopic ossification prophylaxis.

### Case 1: Bilateral fibrous ankylosed hip with fixed kyphotic deformity

**Figure 1:**
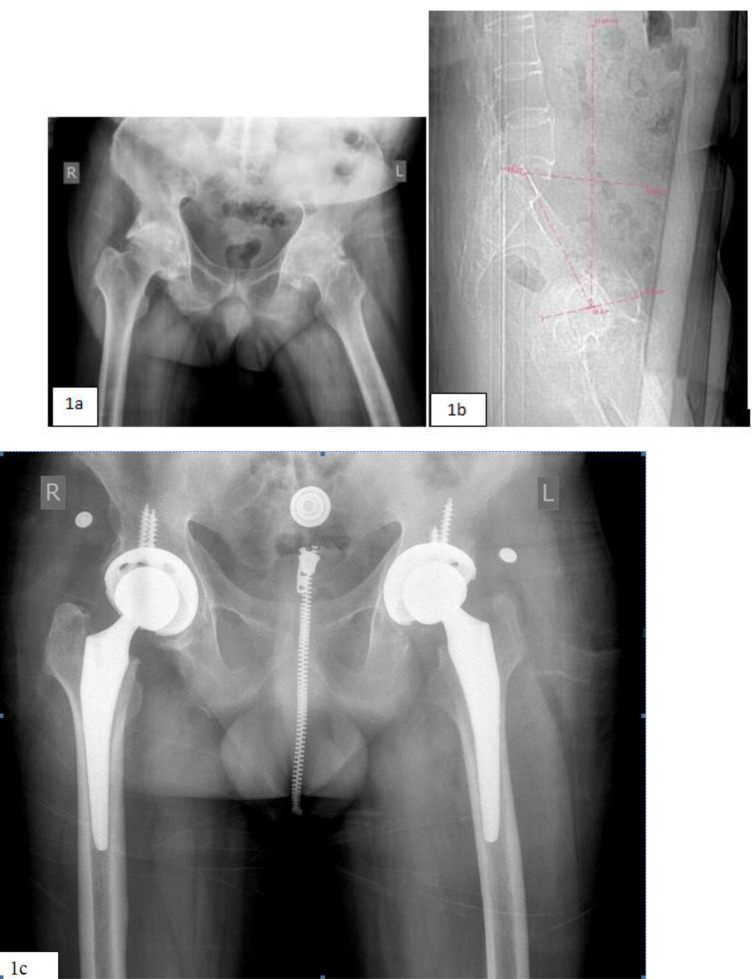
Case 1. (a) Preoperative antero-posterior (AP) views of bilaterally fused hips. (b) Pelvic hyperextension calculation. (c) Six months postoperative antero-posterior (AP) view of the pelvis.

A 40-year-old man presented with bilateral hip ankylosis and fixed kyphotic deformity. His right hip was fused in 60° of flexion and 10° of external rotation. His left hip was fused in 15° of flexion, 30° of abduction, and 20° of external rotation (Figure 1a). Indications for surgery were increasing pain and limitation of activities of daily living. Preoperative radiographs and CT scans showed fibrous ankylosis of both hips and pelvic hyperextension of approximately 26.7° (Figure 1b). He underwent bilateral RTHA in a single session, with the left RTHA performed first.

Preoperative CT-based planning indicated optimal fit with a 56 mm cup (Trident^®^, peripheral self-locking [PSL] version; Stryker, Mahwah, NJ, USA). Cup placement was planned for 36° and 16° of abduction and anteversion, respectively, given the hyper-extended pelvis. We achieved 38° abduction and 17° anteversion, respectively. We dislocated the hip and conducted reaming with a single 54 mm reamer, allowing 2 mm anterior, posterior, and medial walls to be reamed. The robotic arm’s haptic guidance ensured the correct reaming depth and direction. The cup was thereafter impacted under robotic guidance to achieve the desired anteversion and abduction. No fluoroscopy was used. Preoperative CT-based planning indicates optimal fit of size 4 stem as mediolateral fill ratio and the canal fill ratio were considered. The femoral canal was prepared manually, broached serially, and fixed with a cementless stem (size 4 with 127° offset; Accolade II, Stryker) with a 36 mm +0 mm ceramic head. The right hip was treated similarly to the left hip, with a 56 mm cup, 36° and 16° of abduction and anteversion planned. We achieved 38° and 17°, respectively. The right femoral canal was prepared manually, broached serially, and fixed with a cementless stem (size 4 with 127° offset; Accolade II, Stryker) with a 36 mm +0 mm ceramic head. The cup was additionally secured with two screws on both sides.

The patient was mobilized (full weight-bearing with the assistance of a walking aid) within 12 h. There were no complications at the 20-month of follow-up (Figure 1c). The flexion range was 0–90° on both sides, and the patient could walk unaided on level ground at the last follow-up.

### Cases 2–4: Bilateral fibrous ankylosed hips without spinal deformity

Three AS patients (six hips) with bilateral spontaneous hip fusion were included in this group (Table 1). The mean age was 22.1 years, and all patients had pain and functional limitations. Preoperative radiographs and CT scans showed fibrous ankylosis of both hips.

**Table 1:** Characteristics of cases 2–4.

Case no.	2		3		4	
Side	Right	Left	Right	Left	Right	Left
Age (years)	17		22		28	
Gender	Male		Male		Male	
Diagnosis	AS with stiff spine and bilateral fused (fibrous ankylosis) hips		AS without spinal involvement and with bilateral fused (fibrous ankylosis) hips		AS without spinal involvement and with bilateral fused (fibrous ankylosis) hips	
Preoperative fusion position	30° flexion, neutral rotation	30° flexion, 15° abduction, 10° external rotation	10° flexion, 10° external rotation	10° flexion, 10° external rotation	10° flexion, 10° abduction, 15° external rotation	15° flexion, 15° external rotation

RTHA was conducted in a single session in all three cases. Cup abduction and anteversion were planned using CT-based templates. Mako Express Workflow was used (Table 2). A posterolateral approach was used, and reaming was conducted with a single robotic-guided reamer. No fluoroscopy was used. All patients were mobilized (full weight bearing) within 12 hours. The cases were followed for eighteen months (case 2) and sixteen months (cases 3 and 4). We did not find nerve palsy (femoral or sciatic), heterotopic ossification, any episode of dislocation, implant breakage, pulmonary embolism, etc.

**Table 2:** Key RTHA details.

Case no.	2		3		4	
Side	Right	Left	Right	Left	Right	Left
Femoral neck osteotomy	Single	Single	Single	Single	Single	Single
Reamer size (mm)	52	52	56	56	44	44
Acetabular component size (mm)	54	54	54	54	46	48
Bearing	Uncemented ceramic on X3 polyethylene	Uncemented ceramic on X3 polyethylene	Ceramic on X3 Rim Fit cemented cup	Ceramic on X3 Rim Fit cemented cup	Ceramic on X3 Rim Fit cemented cup	Ceramic on X3 Rim Fit cemented cup
Supplementary screws	Nil	2	Nil	Nil	Nil	Nil
Intraoperative fluoroscopy	None	None	None	None	None	None
Operation time (min)	53	55	60	60	65	68
Cup anteversion (planned/achieved)	25°/25°	25°/25°	20°/20°	20°/21°	20°/22°	20°/21°
Cup abduction (planned/achieved MAKO Surgical result	40°/39°	40°/39°	40°/41°	40°/42°	40°/41°	40°/42°
Postoperative cup abduction/cup anteversion (CT based)	40.2°/23.3°	38.7°/23.7°	40.2°/21.3°	43.2°/22.6°	40.6°/21.5°	40.1°/22.3°
Dorr classification (femoral canal)	Type B	Type B	Type A	Type B	Type B	Type B
Canal fill ratio (CFR)	0.53	0.52	0.53	0.51	0.52	0.53

### Case 5: Unilateral bony ankylosed hip

**Figure 2:**
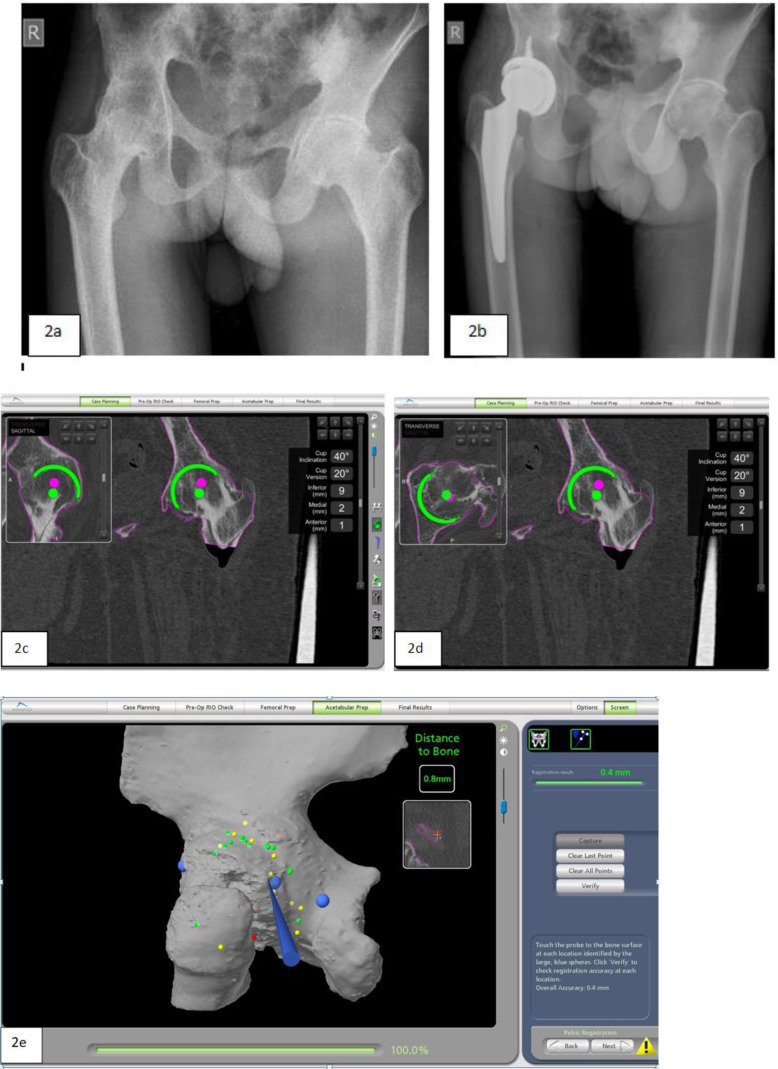
Case 5. (a) Preoperative antero-posterior (AP) view (bony ankylosis of right hip). (b) Six months antero-posterior (AP) view of the pelvis. **(**c) and (d) Preoperative planning for case 5 in transverse and sagittal plane. (e) Verification of pelvic rotation by localizing the anterior superior iliac spine with the hip probe.

A 21-year-old man presented with AS with right hip bony ankylosis fixed in 10° of flexion, 10° of external rotation, and 5° of adduction. Indications for surgery were a sub-optimally fused position and functional impairment (Figure 2a). Preoperative CT-based planning indicated an optimal fit with a 52 mm acetabular cup and a size 5 cementless stem (132° offset; Accolade II, Stryker). The standard posterolateral approach was used. The acetabulum and proximal femurs were registered en bloc without dislocating the hip. Cup placement was planned for 40° of abduction and 20° of anteversion. There was a significant deviation from the standard protocol (requiring authorization from the surgeon) in the bony ankylosed hip, as the surgeon could not dislocate the hip. Such cases need in situ registration before osteotomy. The acetabulum, along with the femoral head and neck, is registered en bloc. The three acetabular alignment points should be as accurate as possible to ensure correct rotational alignment with the virtual model. The subsequent 32 registration points should be widely distributed over the ilium, femoral neck, and the ischium and registered over the hard cortical bone (Figure 2e). Afterwards, the eight verification points are taken to confirm the rotational alignment; while doing so, the surgeon can position the probe over the anterior superior iliac spine to verify the rotation (Figures 2c and 2d). Femoral neck osteotomy is conventionally performed under fluoroscopic guidance to avoid iatrogenic injury to the anterior column. With the Mako robotic system, femoral neck osteotomy was completed under the guidance of the hip probe on the verification screen. Thereafter, the femur was displaced anteriorly to expose the acetabulum. Next, a single robotic-guided 50 mm reamer was used to concentrically ream the entire femoral head down to the planned true acetabulum. The Mako robotic system is semi-automated, with the surgeon maintaining control of the arm. It operates in line with planned haptic boundaries. If the surgeon deviates from the plan, the system will provide auditory and haptic feedback and ultimately shut off the robotic arm. In cases involving fusion, where much attention is required to identify the correct cup placement, the robotic system can provide accurate three-dimensional localization, simplifying this critical step. A 52 mm cup was impacted, with controlled medialization, using the robotic arm. The impacted cup position was fixed at 40° and 20° of abduction and anteversion, respectively. The femur was prepared manually, broached serially, and fixed with a cementless stem (size 5 with 132° offset; Accolade II, Stryker) with a 36 mm −5 mm ceramic head to produce a leg length of 4 mm longer and a combined offset of 0 mm compared to the opposite hip. Intra-operatively, iliopsoas was released from a lesser trochanter, and postoperative adductor tenotomy was also performed. The patient was mobilized (full weight-bearing with assistance) within 12 h. There were no complications at the 16-month follow-up (Figure 2b).

In one patient out of nine joints where bony ankylosis was present, there was evidence of abductor weakness as elicited by a positive Trendelenburg test which was improved, and abductor function restored gradually after having supervised abductor strengthening exercises by the end of 12 months.

Centre of hip rotation has been planned on the MAKO software and accordingly adjustment of size and position of the cup with stem type. The position and angle of the acetabular shell were placed as planned preoperatively on the MAKO, cup position and alignment with canal filling ratio were compared in the postoperative CT scan and immediate postoperative X-rays (Table 2) [3].

## Discussion

To the best of our knowledge, this is the first case series on robotic arm technology used in single-session bilateral RTHA of fused hips in AS patients. Hip involvement occurs in 30–50% of AS patients, 90% of whom have bilateral involvement.

THA in AS patients can be technically challenging, especially in patients with fused hips. Problems increase with bilateral hip involvement, as the opposite hip may hinder patient positioning. There may also be an error in determining the proper cup placement. We conducted bilateral THA in single sessions as staged procedures would take longer to restore mobility and independence. Bilateral THA has been reported as safe and effective for advanced hip disease in AS, with significant improvements in objective outcome measures such as Harris Hip Score (HHS) and patient mobility [4]. Robotic arm-assisted surgery simplifies these complex surgeries by assisting in three-dimensional planning, precise acetabular reaming, component fixation, and real-time intra-operative feedback. RTHA allows accurate preoperative planning and component positioning than conventional THA, and a better implant alignment will give long-term implant survival and outcomes. RTHA has a certain advantage over conventional THA, including planned and intra-operative complication rates.

Difficulties regarding performing THA in AS patients can be categorized as patient positioning, surgical exposure, femoral neck osteotomy, identification of the true acetabulum, and proper cup positioning [1]. Difficulties regarding exposure are associated with the fusion position and surgical approach used. It is difficult to use a posterolateral approach when the hip is fused in an externally rotated position, as the greater trochanter obscures the femoral neck posteriorly, making in situ osteotomy challenging. Exposing the femoral neck via an antero-lateral approach is a viable option in these cases. Femoral neck osteotomy is challenging, and fluoroscopic guidance is often required. With robotic guidance, the hip probe provides real-time feedback on the level, direction, and location in relation to the femoral neck osteotomy. Marks can be made using diathermy prior to osteotomy. The robotic system eliminates the need for excessive exposure and trochanteric osteotomy, which increases the incidence of heterotopic ossification [5, 6]. In our case series, we showed the fusion position varies in different patients (Table 1). We performed all RTHAs using the posterolateral approach under robotic guidance. We managed to dislocate the hip in all cases except one (involving bony ankylosis), in which en bloc registration was conducted. After adequate exposure, the navigation tool was used to complete registration. The mean registration time was 11 min (range: 5–25 min). After registration, the robotic arm was used for reaming as planned under hepatic guidance, which helped prevent medial wall breach and ensured the desired cup placement. We managed all cases with a single reamer, and stable fixation was achieved. The robotic system enabled the identification of the true acetabular plane and anatomy, which are generally distorted in ankylosed hips. Lastly, the preoperatively planned cup placement was achieved (with variation ≤ 2°) in all cases. RTHA thereby shows promising results regarding cup placement.

Sagittal pelvic malrotation can be present in AS patients. Pelvic hyperextension compensates for kyphosis. This increases the cup anteversion and abduction angles during walking [7]. Rivière et al. [8] recently described the concept of personalized kinematically aligned THA, in which cup position is determined not by the traditional Lewinnek “safe zones” but by the functional cup orientation. We optimized the abduction and anteversion angles with sufficient bone coverage. Another common problem is the poor quality of the acetabular bone, likely because of the disuse of osteopenia and medication. Over-reaming in these cases can compromise the acetabular walls. Conventional acetabular cup orientation techniques include free-hand placement, external alignment guides and anatomic bony and soft tissue landmarks, and combined techniques – including preoperative radiographic templating, intra-operative stability assessment, and intra-operative radiographic evaluation. These techniques have been repeatedly shown to lead to cup mal-positions rates of ≥50% [9]. This is because of poor visualization, the wide variation in the position of anatomic landmarks among patients, variation in intra-operative patient position with resulting mechanical guide imprecision, inaccurate templating, and imprecise radiographic interpretation. RTHA helps to eliminate all these challenges.

Understanding the hip–spine relationship is important before an ankylosed hip replacement. In healthy individuals, the lumbar spine is flexible in the sagittal plane. The pelvis tilts posteriorly to accommodate hip flexion when moving from standing to sitting [10]. For each degree of increased pelvic tilt, acetabular anteversion increases by 0.7–0.8°. Lumbar spine stiffness can prevent this normal accommodation and lead to anterior impingement when sitting or posterior impingement when standing. In these cases, increased anteversion of the acetabular component is required to compensate for the reduced posterior tilt imposed by the stiff spine.

AS with severe fixed kyphotic deformity increases the risk of anterior dislocation of the prosthesis. Patients with fixed kyphosis tend to hyperextend their hips when standing upright to look forward. If the cup is inserted according to the acetabulum anatomy, it becomes abnormal when the patient stands upright. Pelvic hyperextension brings the cup to a more open position with exaggerated anteversion. Surgical management of these cases primarily involves hip arthroplasty and – less frequently – spinal osteotomy to correct the kyphotic deformity [11], and occasionally both.

Regarding coronal-plane pelvic deformities with bilateral hip involvement in which the contra-lateral hip has a fixed abduction or adduction deformity, using conventional methods to determine the true acetabular abduction will cause errors because of the external references used [12]. Surgeons need not be concerned about the superior or inferior tilt of the pelvis during cup positioning when using the robotic arm. This is because the robotic system takes references directly from the bone and provides real-time cup position data during cup reaming and impaction, regardless of patient position. Thorough CT-based planning and enhanced robotic-guided precision enabled us to treat these challenging cases (using Mako Express Workflow, as the latest software, version 4, was not yet available). Patients with juvenile AS who present with more peripheral joint involvement (clinically and radiologically) are more likely to require arthroplasty [13]. Restricted axial mobility [14] and limited hip flexion and extension are also risk factors [15].

In our case series, the mean blood loss was 550 ml, and the operation time ranged from 51 to 70 min, including registration [16].

Swanson and Huo [17] have reported that the overall clinical outcome is generally less satisfactory than THA performed to treat osteoarthritis and other etiologies. In comparison to conventional THA in ankylosed hips, immediate postoperative range of motion (ROM) and limb length were just as satisfactory as routine THA. In all these cases, dislocation is anticipated due to pelvic malrotation and soft tissue imbalance, so a larger head and dual mobility head are preferable.

The limitations of the robotic arm technology include the requirement for CT scans (which involve patient exposure to radiation), higher surgical costs compared to conventional THA, and the need for additional training and expertise. We did not do any functional scores or measured range of movement (ROM) after the operations, but the alignment was measured as this was our primary outcome in this study. There were no control groups to prove the superiority of robotics in ankylosed hips. Although we observed promising early clinical and radiological outcomes, studies of long-term outcomes are warranted.

## Conclusion

Robotic arm technology helps surgeons provide real-time information on the relative position of the femur, pelvis, instruments, and implants to guide surgery and accurate implant placement. This thereby decreases variation in acetabular cup orientation and improves the surgeon’s ability to determine changes in leg length, offset, and version. As such, the goals of improving physiologic biomechanics and decreasing complications and revision surgery – which are fairly common in complex cases of ankylosed hips – may be achieved.

By improving precision and dexterity, robotic arm technology has simplified every stage of complex fused hip replacement, which is difficult to conduct using conventional THA methods.
